# Skin graft surgery and its impact on platelet counts in Iranian burn patients: a non-randomized clinical trial

**DOI:** 10.1186/s12893-024-02489-x

**Published:** 2024-07-03

**Authors:** Jafar Kazemzadeh, Shiva Pakzad, Naser Parizad, Yashar Jafari

**Affiliations:** 1 Reconstructive and Burn Surgery Department, Urmia University of Medical Sciences, Urmia, Iran; 2 Childhood Obesity Research Center, Urmia University of Medical Sciences, Urmia, Iran; 3 Nursing and Midwifery Faculty, Campus Nazlu, 11 KM Road Seru, Urmia, 575611-5111 West Azerbaijan Iran; 4 Department of General surgery, Urmia University of Medical Sciences, Urmia, Iran

**Keywords:** Skin graft, Surgery, Platelet count, Autologous transplant, Iran

## Abstract

**Background:**

Platelets are critical in maintaining homeostasis and immune response in burn patients. The concentration of platelets decreases in burn patients, and any intervention that increases serum platelet concentration can prevent serious consequences and patient death. The present study aimed to assess the impact of skin graft surgery on burn patients’ platelet counts.

**Methods:**

In this non-randomized clinical trial, 200 burn patients were investigated. The patients were recruited from the surgical ward of Imam Khomeini Teaching Hospital during the first six months of 2021. After completing the checklist, patients underwent skin graft surgery. Blood was taken from the patients during surgery in the operating room and on the third and fifth day after the surgery to check platelets. Data analysis was conducted using SPSS software (ver. 22.0).

**Results:**

Most patients (63.5%) were male, and 73 (36.5%) were female. One hundred eighty-one patients (90.5%) had deep burns, and 19 (9.5%) had superficial burns. The mean burns percentage in the patients was 19.3 ± 15.4%, the lowest was 2%, and the highest was 90%. The most common burns were caused by flame (42%) and boiling water (30.5%). The patients’ outcomes revealed that 6% gained complete recovery, 86.5% partial recovery, 2.5% showed transplant rejection, and 5% died. Mean platelet levels in deceased patients had an upward trend. The mean platelet counts of patients were elevated during surgery (289,855 ± 165,378), decreased three days after surgery (282,778 ± 317,310), and elevated again five days after surgery (330,375 ± 208,571). However, no significant difference was found between the mean platelet counts during surgery, the third and fifth days after surgery in patients undergoing skin grafts (*P* = 0.057).

**Conclusions:**

This study suggests that skin graft positively increases the patient’s platelets. Further studies are needed to confirm the findings and elucidate the mechanism. Iranian Registry of Clinical Trial approval code (IRCT# IRCT20131112015390N8 & 06/01/2024).

## Background

Burn wounds are a common cause of skin damage that can lead to bleeding, dehydration, and infection. Quick healing is critical to preventing complications [1]. Burn wounds typically stall in the inflammatory phase, preventing progression towards wound healing. This leads to secondary complications, prolonged treatment, and increased costs [1, 2]. One of the most critical challenges in medicine is achieving complete skin repair in the shortest time with the least complications. This requires extensive research on the wound-healing process and related factors [3]. Skin grafting effectively treats burns as it covers the affected area and allows for regeneration [3, 4].

Skin grafts help in wound healing by replacing skin collagen, creating a biological barrier, and protecting the wound [5, 6]. The grafted skin initially attaches to the grafting site and turns red on the fourth day. It gradually returns to its natural color by the eighth day and may take up to three weeks to become indistinguishable from normal skin [6–8]. The graft is secured by the plasma naturally secreted from the uncoated area, which causes fibrin formation. Over time, vascular buds start to penetrate deeper into the graft and promote skin tissue growth. In the next stage, the adaptation stage, the graft begins to resemble normal skin. Sensations such as pain, heat, and touch may start to be felt at the transplant site between six to twelve months after the surgery [6, 7].

There are several methods for skin grafts [9], but skin autograft tends to be more effective in healing wounds [10]. To achieve the desired outcome, it’s important to prevent bleeding and the formation of blood clots under the graft. Properly performing the grafting process and dressing the area with gentle pressure can help prevent hematoma formation and infection at the transplant site [3]. The recovery process of full-thickness skin graft involves three stages: plasma uptake, inoculation, and capillary growth [11]. When a graft is placed on a weak vascular network, it is likely to develop ischemia. The absorption phase is crucial to keeping the graft alive and helping it gain weight. After 48 h, a delicate vascular network is formed in the fibrin layer between the graft and the recipient substrate, which helps the graft survive and grow [12]. Platelet-enriched plasma triggers inflammatory reactions in the full-thickness skin graft and the receptor site, which ultimately facilitate the graft’s attachment at the receptor site. These reactions also promote the development of new blood vessels within the graft by releasing growth factors [13].

The wound healing process is regulated by various growth factors such as epidermal growth factors (ETFs), fibroblast growth factors (FGFs), transforming growth factors (TGFs), and insulin-like growth factors (IGFs) [14]. In normal skin, dermal and epidermal cells secrete insulin-like growth factors. However, during skin damage, such as cuts, burns, or wounds, most epidermal cells, including macrophages and platelets, secrete these growth factors. This group of growth factors stimulates mitogenic fibroblasts and is involved in angiogenesis [6, 15]. Previous study has shown that IGFs and platelet-derived growth factor (PDGF) play a crucial role in wound healing. They increase the thickness of both dermis and epidermis [6].

The mechanism behind the effect of platelets on transplantation has yet to be fully understood [13]. Platelets play a dual role in connective tissue and patient health. They are essential for primary hemostasis, tissue repair, and regeneration but also contribute to ischemic injury and inflammatory processes [16]. Platelets play a vital role in facilitating angiogenesis and tissue proliferation. Therefore, to ensure a successful transplant, it is essential to study the immune system and the factors involved in it through various methods. This can help identify strategies to improve the chances of success [17]. Concentrations of plasma platelets are crucial for burn patients’ recovery and outcome [18]. Studies have reported a significant correlation between low platelet concentrations in patients’ plasma and acute renal failure and septic shock [18, 19]. Thus, the platelet concentration in plasma can be used to indicate the burn patient’s prognosis. [18]. Given the crucial role of platelets in burn patients, the decrease in platelet concentration can lead to severe consequences and even death. Therefore, increasing serum platelet concentration can help prevent such outcomes [18, 20]. Hence, the present work was conducted to evaluate the effect of skin grafts on patients’ platelet counts after surgery. We hypothesized that skin grafting might affect platelet counts in burn patients.

## Methods

This study was a non-randomized clinical trial study with a single arm, own control and before and after design. It was conducted in Imam Khomeini Teaching Hospital in Urmia, Iran during the first six months of 2021. This study was approved by the hospital’s research council and the ethics committee of Urmia University of Medical Sciences. It was also registered in the Iranian Registry of Clinical Trials^1^ (IRCT# IRCT20131112015390N8 & 06/01/2024) (See Related files). The CONSORT 2010 checklist was used to ensure quality reporting in the present study (See Related files).

### Sampling

**Fig. 1:**
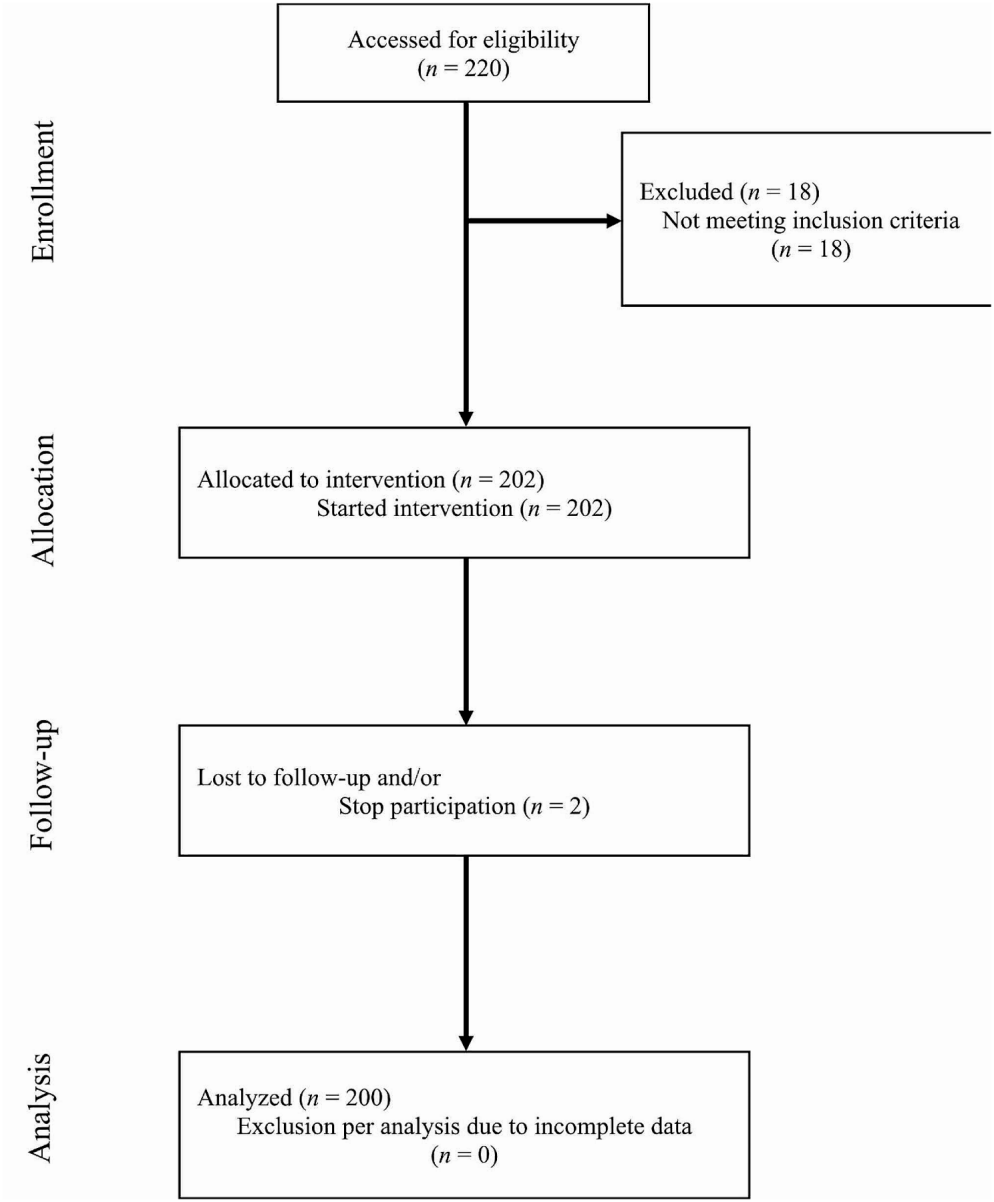
Modified Consort flow diagram for a single arm, non-randomized, own control study

Using Stata statistical software, the minimum sample size required was estimated to be 200 people, with a significance level of 5%, power of 80%, and ES of 0.186. Considering a 10% sample attrition, 220 samples were considered for the study. Patients referred to the burn surgical ward were selected using convenience sampling. The inclusion criteria included having no history of smoking, diabetes, or any signs or symptoms of sepsis or skin infections (Temp < 37° C orally) and not using any drugs that affect platelet factors. A total of 220 patients were enrolled in the study, but 18 were excluded because they didn’t meet the inclusion criteria. Fourteen of the excluded patients were smokers, three had type II diabetes, and only one was taking aspirin. Two patients were excluded from the study due to their lack of cooperation during the study. The remaining 200 patients were analyzed (Fig. 1).

### Intervention

Eligible patients received skin grafting by the burn surgeon (first author) within the first week of hospitalization. A checklist was used to collect demographic information such as age, sex, and clinical information such as severity, percentage, cause of burns, platelet count, type of transplant, patient outcome after skin grafting, and patient’s condition at discharge. Blood samples were collected from patients to determine their platelet count during surgery in the operating room and on the third and fifth days after transplantation (Primary outcome). The number of platelets in each blood unit was recorded as 1000 per cubic millimeter. The patient’s condition was evaluated at discharge based on the wound healing rate (stable vital signs and lack of sepsis criteria) and transplant success (complete graft fixation to the wound bed). Before the study, the patients were informed, and written consent was obtained. The study was completed on June 30th, 2021, after analyzing 200 patients. Furthermore, no instances of physical or psychological harm were reported throughout the study.

#### Skin graft surgery

The surgeon performed surgical debridement and skin graft to repair the burn site in patients with relatively profound burns that penetrate the reticular dermis layer, blood vessels, and skin appendages. For this purpose, all patients had graft surgery in one session after the patient’s initial resuscitation. However, patients were debrided several times based on the TBSA burn percentages. Patients with less than 40% burn were debrided in one session, while those with over 40% were debrided in two or three sessions. In burn patients, a surgical procedure is performed to remove ischemic and necrotic tissue layer by layer using a dermatome. This tissue increases the chance of infection and is the most important cause of death among burn patients. When the wound began to bleed, it indicated blood supply and no involvement in a burn. After stopping the bleeding, healthy skin grafts were taken from areas of the body that were not affected by burns. The thighs are the best areas for skin grafts because of the amount of skin in the area. If the thighs were also affected, the grafts were taken from other regions, such as the legs, arms, forearms, and trunk. The Dermatome was used to adjust the depth of skin removal, which involved using a semi-thick skin graft (SSG) or a full-thick skin graft (FSG), depending on the case. The skin was removed from the area requiring treatment, which was then debrided, placed, and sutured using a stapler. After the graft operation, a sterile vaseline gauze was placed over the grafted area and left undisturbed for five to seven days. The remaining skin helped ensure that the graft was successful. Skin grafting involves transplanting healthy skin from one part of the body to another to repair deep burns. This technique reduces the risk of infection, a significant concern for burn patients, and increases their chances of survival. Early excision and grafting have revolutionized the treatment of burn patients and significantly improved their chances of recovery. Most burn centers can save patients with up to 50% burns by performing excision and skin grafting.

### Data analysis

The data was analyzed using descriptive statistics such as frequency, percentage indices, and graphs. The Normality of the data was determined using the Smirnov-Kolmogorov test. Changes in mean platelet count during surgery were compared with the third and fifth days of skin grafting using repeated measures ANOVA. Data were analyzed using IBM SPSS Statistics for Windows, version 22.0 (IBM Corp., Armonk, N.Y., USA). The significance level was considered less than 0.05.

## Results

The results showed that 63.5% of patients receiving skin grafts were male and 36.5% female. The mean age of patients was 33.7 years, of which 21% were between 30 and 39 years, 18.5% were between 20 and 29 years, and 18.5% were between 40 and 49 years. Regarding the cause of burns, 42% of patients burnt due to the flame, and 30.5% had burns due to boiling water. The results also showed that 90.5% of the patients suffered from deep burns, while 9.5% had superficial burns. When the outcome of patients who received skin grafts was analyzed, it was observed that 6% of patients had fully recovered, 86.5% had partially recovered, 2.5% rejected the implant, and 5% died (Table 1).

**Table 1:** Frequency distribution and percentage of patients’ information receiving skin grafts

Variable		*N*	%
Gender	Male	127	63.5
	Female	73	36.5
Age	< 10 years	24	12.0
	10–19 years	20	10.0
	20–29 years	37	18.5
	30–39 years	42	21.0
	40–49 years	37	18.5
	50–59 years	20	10.0
	60–69 years	17	8.5
	≥ 70	3	1.5
Cause of burns	Fire flame	84	42.0
	Boiling water	61	30.5
	Explosives	6	3.0
	Hot bitumen	5	2.5
	Hot liquid	8	4.0
	Gas explosion	19	9.5
	Electricity	13	6.5
	acid	4	2.0
Types of burn	Full-thickness burns	181	90.5
	Superficial burns	19	9.5
Skin graft outcome	Complete recovery	12	6.0
	Partial recovery	173	86.5
	Rejection	5	2.5
	Death	10	5.0

Table 2 shows the mean and standard deviation of patients’ age, percentage of burns, and the duration of hospitalization before surgery. The hospitalization period before surgery ranged from two to seven days (4.09 ± 1.198) (Table 2).

**Table 2:** Burn percentage, age, and hospitalization days of patients before surgery

Variables	M ± SD	Median	Minimum	Maximum
Age (Years)	33.7 ± 17.7	33	1	76
Burn percentage (%)	19.3 ± 15.4	16	2	90
Hospitalization days before surgery (Days)	4.09 ± 1.198	4	2	7

The results showed that ten deceased patients’ platelet counts were high during surgery and on the third and fifth days after the procedure. The effect size between the mean platelet counts of the deceased patients during surgery and on the fifth day after surgery was 0.2 (Table 3).

**Table 3:** The mean platelets level during surgery, on the third and fifth days after surgery in deceased patients

	Patients’ platelets level		
	During surgery in the operating room	The third day after surgery	Fifth day after surgery
	M ± SD	M ± SD	M ± SD
Death patients	405,400 ± 236,949	443,700 ± 231,207	507,700 ± 25,206

**Fig. 2:**
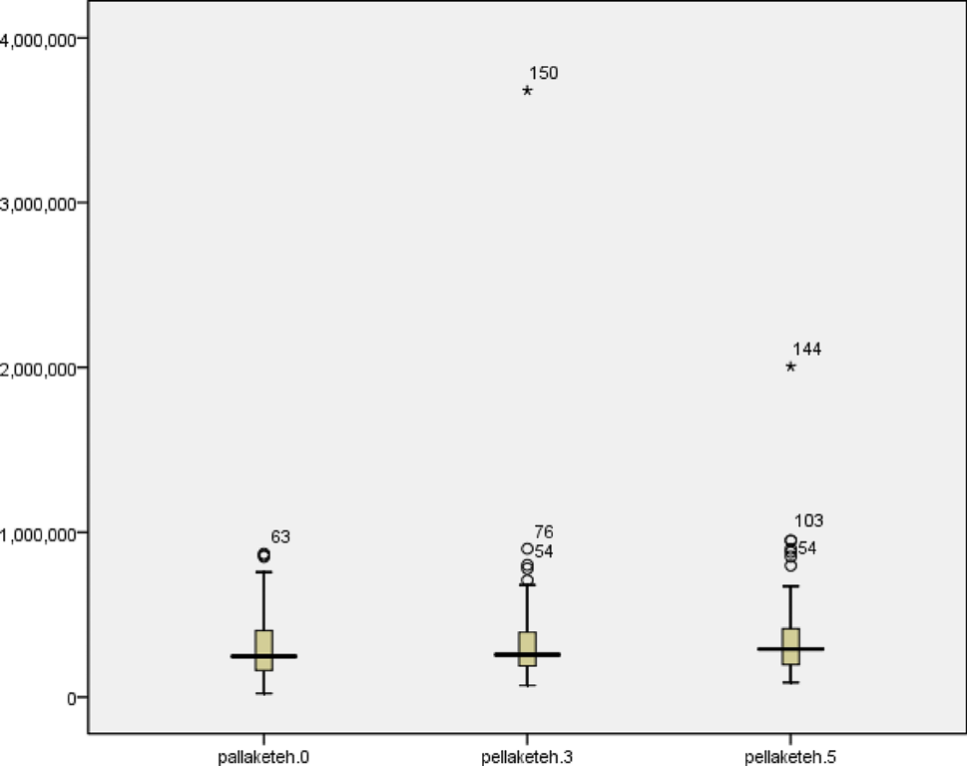
Mean platelet level during surgery and third and fifth days after surgery in burn patients

The mean platelet counts decreased on the third postoperative day and increased again on the fifth day after the surgery in surviving patients. The effect size for the difference between the patient’s platelets during surgery and on the fifth day after surgery was 0.2 (Table 4) (Fig. 2).

**Table 4:** The mean platelets level during surgery, on the third and fifth days after surgery in alive patients

Variables		M ± SD	Median	Minimum	Maximum
Patients’ platelets level	During surgery in the operating room	289,855 ± 165,378	247,500	22,100	870,000
	Third day after surgery	282,778 ± 317,310	257,500	71,000	3,680,000
	Fifth day after surgery	330,375 ± 208,571	290,000	90,000	2,005,000

The results show that the significance of F was 0.057, which is considered insignificant. Therefore, no significant difference was observed in the mean platelet counts during surgery and on the third and fifth days after surgery in patients who underwent skin grafts (Table 5).

**Table 5:** Tests of within-subjects effects for platelet level in patients receiving skin grafts

Variable	Epsilon	Mean square	F	Sig.
Platelet count	Sphericity Assumed	85542455850.001	2.887	0.057
	Greenhouse-Geisser	108233200780.141	2.887	0.070
	Huynh-Feldt	107516792148.232	2.887	0.069
	Lower-bound	171084911700.002	2.887	0.091

## Discussion

The study aimed to evaluate the effect of skin grafts on post-surgery platelet count in burn patients. Most participants were males (63.5%), with a mean age of 33.7 years. The study found that the age range with the highest number of accidents was 30–39. About 90.5% of the burns were full-thickness, and after skin graft surgery, 86.5% of the patients had partially recovered, and 5% had died. According to Kolaei et al.‘s study, most burn patients were males (69.6%) between the ages of 20 and 39, with a mean age of 32.95. The most common type of burn experienced by these patients was full-thickness burns, which is consistent with the current study’s findings. In the present study, flame, and boiling water were the first and second causes of burns. In most studies, boiling water was reported to be the most common cause of burns [21, 22].

Few studies have investigated changes in platelet counts over time in burn patients. Takashima et al. observed that platelet counts decreased within 7–12 days, returned to normal range, and increased significantly before stabilizing after two months [23]. Our study’s time-dependent changes in platelet count are similar to the platelet counting trend in the studies by Sarda et al. [24] and Bartlett et al. [25]. In Bartlett’s study, patients with severe burns had a peak in thrombocytopenia on the third day. This was followed by a return of platelet counts to baseline levels within the first week, and then a significant increase [25]. In Warner et al.‘s study, platelet count significantly increased on day seven compared to day four and on day 14 compared to day seven [26], which was in line with our study. In this study, the platelet count significantly increased on day five compared to day three and during surgery, possibly due to platelets contributing to the inflammatory response to burn injury.

The pathophysiological changes caused by burn injury are not limited to the specific area of injury. The inflammatory response throughout the body is due to the effect of cytokines and endotoxins. Increased platelet counts are a natural response to various inflammatory stimuli. However, thrombocytopenia is a significant concern in critically ill patients and is thought to play a crucial role in worsening the disease process [26]. Previous studies have found a correlation between low platelet count in critically ill patients and reduced survival rates [18, 27]. Studies have confirmed the crucial role of platelets in predicting burn patients’ prognosis [28, 29]. Sen et al. reported that severe thrombocytopenia is a risk factor and predictor of mortality in patients with severe burns [29]. Thus, the serial platelet count can be a prognostic indicator for burn patients during recovery. In patients with moderate or severe burns, frequent platelet counts are necessary during this period to increase platelets before discharge. Hence, whenever platelet counts begin to decline, all measures should be initiated to support the burn patient’s general condition, including injection of intravenous fluids and antibiotics, optimal care of burn wound, debridement, or extraction and transfusion.

In our study, deceased patients had significantly higher platelet counts throughout hospitalization, in contrast to the findings of Pavić et al.,28 study [28]. They indicated that platelet counts were significantly lower in patients who died throughout the hospitalization except on the first day. Cato et al. found that platelet counts were significantly lower in deceased patients at all controlled time points, except on the first day, when platelet counts in this group of patients were significantly higher than those who survived [27]. In El-Sonbaty et al.‘s study, the deceased patients had significantly lower platelet counts than the surviving patients [30]. These results were in contrast to our findings. A plausible reason could be dehydration, inflammation, and infections in our deceased patients.

El-Sonbaty et al. reported that platelet counts increased significantly in alive patients after burn injury on day 7 [30], which is consistent with our study’s findings. Our study also revealed a gradual increase in platelet count in patients. The literature review shows that thrombocytopenia typically occurs between days two and five, followed by a peak of thrombocytosis around days 11–17. [23, 26, 27, 31]. Either mechanism can cause premature thrombocytopenia: Fluid therapy-induced hemodilution can decrease platelet production or activity following bone marrow suppression. However, it is challenging to determine how much hemodilution affects the rate at which platelets shrink after a burn. Studies have shown that platelet counts continue to decline even after discontinuing fluid therapy [25, 26, 32]. Therefore, it is reasonable to suggest that platelets are consumed inside the burn wound due to the destruction of cutaneous blood vessels and the formation of subsequent microthrombi [33].

It has been well documented that increased vascular permeability may lead to platelet activation and activation of coagulation factors, resulting in subsequent accumulation and consumption. Activated platelets can interact with circulating neutrophils and monocytes, enhancing their ability to migrate to affected areas and affecting the platelet count in the medium [34, 35]. Based on the results obtained, further research is required to understand the platelet count in these patients. Other factors that can affect platelet count, such as medication, must also be considered. For example, drugs like heparin can cause a condition known as heparin-induced thrombocytopenia (HIT). However, in this patient group, HIT is unlikely to be the cause, as it usually starts 5–10 days after heparin use [36]. According to published reports, piperacillin-tazobactam, a commonly prescribed antibiotic, can rarely cause thrombocytopenia in some patients. The peak platelet count in burn patients may be due to increased levels of thrombopoietin (TPO) in circulation after a decrease in total platelet mass early in the injury. This may explain the recurrence of thrombocytosis in patients by triggering the production of platelets in the bone marrow. The post-injury systemic inflammatory response syndrome (SIRS) may worsen due to the presence of inflammatory cytokines such as IL-6 [37, 38]. Individuals may experience increased bone marrow activity due to an inflammatory response. Thus, monitoring the levels of TPO and IL-6 over time can provide valuable information about the megakaryocyte/platelet condition. In addition to thrombocytopenia, the TBSA percentage, age, and the likelihood of respiratory injury are the most critical factors determining mortality after a burn injury [39, 40]. Ten patients (5%) who were significantly older than the surviving patients (with a mean age of 44.3 years) died during the follow-up period.

The mean percentage of burns in patients was 19.3 ± 15.4, and most of them had deep burns. Multiple studies confirm that using autologous platelet-rich plasma (PRP) in wound beds to anchor skin grafts has a significant effect due to its hemostatic, adhesion, and healing properties rather than relying on conventional methods such as sutures, staplers, or adhesives [16, 22, 31]. To the best of our knowledge, no study has been conducted on the effect of skin grafting on blood platelet counts. Our study is the first of its kind in this regard.

### Research limitations

One significant limitation of the study was the failure to report the TBSA for all patients individually and its potential correlation with their platelet count. This oversight could have had a substantial impact on the results. This study was conducted with a relatively small sample size and for a short period following skin graft surgery; therefore, generalizing the results should be cautiously approached. It is recommended to conduct similar studies for longer periods. The success of grafting may depend on various factors, including genetic factors. However, investigating the underlying genetic factors is limited due to the lack of facilities.

## Conclusions

It was observed that the mean platelet counts of patients tended to increase after undergoing skin grafting on the third and fifth days; however, this increase was not considered statistically significant. Therefore, skin grafting may positively affect the patient’s platelet levels. More research is required to understand the mechanism behind it fully. It is recommended that more experimental studies be conducted. These studies should involve larger sample sizes and be carried out for a longer duration to confirm the effect of skin grafting on platelet counts in burn patients and explain its mechanism.

## Data Availability

The datasets generated during and/or analyzed during the current study are available from the corresponding author on reasonable request.

## Footnotes

1 The full trial protocol can be accessed at: https://irct.behdasht.gov.ir/user/trial/74084/view.

## Abbreviations

ETFs: Epidermal Growth Factors
FGFs: Fibroblast Growth Factors
TGFs: Transforming Growth Factors
IGFs: Insulin-like Growth Factors
PDGF: Platelet-derived Growth Factor
ANOVA: Analysis of variance
SPSS: Statistical Package for the Social Sciences
HIT: Heparin-induced Thrombocytopenia
TPO: Thrombopoietin
PRP: Platelet-rich Plasma
